# Barrier and Immune Modulation by *Limosilactobacillus reuteri* ATCC PTA 6127 in Canine Epithelial and Immune Cells Under Lipopolysaccharide Challenge

**DOI:** 10.3390/ijms27125546

**Published:** 2026-06-19

**Authors:** Andreea Cornelia Udrea, Katrine Bie Larsen, Steffen Yde Bak, Niels Christensen, Adrian Schwarzenberg, Akila Rekima, Ashley Hibberd, Chong Shen

**Affiliations:** 1 Gut Immunology Lab, R&D, Health & Biosciences, International Flavors & Fragrances Inc., Edwin Rahrs Vej 38, 8220 Brabrand, Denmark; andreea.cornelia.udrea@iff.com (A.C.U.); katrinebie.larsen@iff.com (K.B.L.); akila.rekima@iff.com (A.R.); 2 Enabling Technologies, R&D, Health & Biosciences, International Flavors & Fragrances Inc., Edwin Rahrs Vej 38, 8220 Brabrand, Denmark; steffen.yde.bak@iff.com (S.Y.B.); niels.christensen@iff.com (N.C.);; 3 R&D, Health & Biosciences, International Flavors & Fragrances Inc., Madison, WI 53716, USA; ashley.hibberd@iff.com

**Keywords:** *Limosilactobacillus reuteri*, postbiotics, tight junctions, epithelial barrier, cytokine regulation, cellular stress response, lipopolysaccharide (LPS), immune modulation, macrophage activation, redox homeostasis

## Abstract

Coordinated responses of intestinal epithelial and immune cells are essential for maintaining barrier integrity and immune homeostasis in dogs, yet our mechanistic understanding of probiotic-derived metabolites remains limited due to reliance on non-canine experimental models, highlighting the need for studies in canine-derived systems. Here, we investigated the effects of metabolites derived from *Limosilactobacillus reuteri* strain ATCC PTA6127 (Lr6127), delivered as a cell-free supernatant (CFS), on canine epithelial MCA-B1 cells and macrophage-like DH82 cells subjected to lipopolysaccharide (LPS)-induced inflammatory stress. Lr6127 CFS significantly reduced epithelial permeability, decreasing FITC–dextran leakage to 94.9 ± 1.9% (normalized relative to LPS-treated control, which was set as 100%) (*p* < 0.001), despite no detectable transcriptional changes in tight junction, adherens junction, or mucin genes. Barrier effects were instead associated with changes in markers of cellular stress responses, with heme oxygenase expression decreasing from 0.9 ± 0.1 to 0.7 ± 0.1 (*p* < 0.05). In DH82 immune cells, Lr6127-derived metabolites altered LPS-induced stress- and inflammation-related gene expression patterns; enhanced anti-apoptotic responses, as reflected by the increased BCL2 expression (1.4 ± 0.1 vs. 1.0 ± 0.0; *p* < 0.01) and elevated BCL2/BAX ratios (*p* < 0.01); and reduced expression of pro-inflammatory mediators including IL-6 and CCL2 (*p* < 0.05–0.001). Proteomic analysis corroborated that Lr6127-derived metabolites reduced the abundance of inflammatory and STAT-associated signaling proteins under LPS challenge, while indicating context-dependent changes in immune-related protein profiles under resting condition. Collectively, these results suggest that Lr6127-derived metabolites improved epithelial barrier function, which was accompanied by coordinated changes in cellular stress-related and inflammatory pathways, highlighting their potential to positively influence host responses.

## 1. Introduction

The canine gastrointestinal tract is a critical interface between dietary antigens, commensal microbiota, and the host immune system and plays a central role in maintaining systemic homeostasis [1,2]. Dogs are routinely exposed to multiple stressors, including dietary changes, environmental stress, dysbiosis, and enteric pathogens, which collectively challenge intestinal barrier integrity [3,4]. Among these insults, bacterial endotoxins such as lipopolysaccharide derived from Gram-negative bacteria are particularly relevant as they activate innate immune signaling and exacerbate epithelial barrier dysfunction [1,5]. Increased intestinal permeability facilitates endotoxin translocation, amplifying mucosal inflammation and contributing to chronic inflammatory enteropathies in dogs [3,6]. Importantly, barrier dysfunction in dogs is being increasingly recognized as a functional and stress-driven phenomenon, rather than a consequence of irreversible epithelial damage [4,6].

Thus, nutritional strategies aimed at supporting intestinal barrier function have gained substantial attention in companion animal research [1]. Probiotics have been widely investigated for their ability to modulate microbiota composition, immune responses, and epithelial function; however, their efficacy is often strain-specific and influenced by viability, host colonization efficiency, and environmental conditions [7,8]. These limitations have motivated research on postbiotics, which are defined as bioactive microbial metabolites or components that confer host benefits independently of live bacteria [9]. Postbiotics offer advantages regarding stability, safety, standardization, and mechanistic clarity, making them attractive alternatives or complements to live probiotics [9,10]. Within this framework, *Limosilactobacillus reuteri* has emerged as a particularly relevant species due to its ability to produce diverse metabolites that modulate immune responses, redox balance, and epithelial function across multiple model systems [11,12].

Despite this growing body of literature, mechanistic insights into probiotic-derived metabolites remain largely extrapolated from non-canine models. Most in vitro studies investigating epithelial barrier function rely on human colorectal carcinoma cell lines, such as Caco-2 or HT-29, or on porcine intestinal epithelial models including IPEC-J2 [4,6]. These systems differ substantially from canine intestinal epithelium regarding differentiation dynamics, innate immune receptor expression, cytokine responsiveness, and regulation of epithelial stress pathways, which can influence the accuracy of barrier integrity assessments [1,6]. Furthermore, species-specific differences in Toll-like receptor signaling, sensitivity to lipopolysaccharide-induced inflammation, and epithelial barrier regulatory mechanisms limit the translational relevance of such models for canine intestinal health [4]. This includes the probiotic or postbiotic effects inferred from human or porcine epithelial systems, underscoring the need for canine-derived epithelial and immune models.

A further limitation of the existing literature is that many studies evaluate barrier integrity using transepithelial electrical resistance or macromolecular flux assays in human or porcine cell lines and subsequently infer mechanisms based exclusively on changes in tight junction gene expression [4,13]. However, epithelial permeability can be markedly influenced by cytoskeletal tension, cellular stress responses, redox balance, and immune-derived mediators, even in the absence of detectable alterations in tight junction gene transcription [6,14]. This issue is particularly relevant in dogs, where inflammatory stress, rather than constitutive junctional defects, appears to underlie barrier dysfunction in many clinical contexts [3,15]. Altogether, the reliance on non-canine epithelial models and a tight-junction-centric interpretative framework risks oversimplifying barrier regulation and obscuring species-specific mechanisms critical to canine gut health.

In the present study, we employed canine epithelial MCA-B1 cells and macrophage-like DH82 cells to investigate the biological effects of *Limosilactobacillus reuteri* strain ATCC PTA 6127 (Lr6127). Our group uses live bacteria and cell-free supernatants in parallel experimental frameworks to disentangle direct microbial effects from those mediated by bacterial metabolites. The present study specifically focused on the effects of cell-free supernatants; previous complementary studies by our group have evaluated live *L. reuteri* under comparable conditions. Here, the use of cell-free supernatants enabled the targeted investigation of metabolite-driven mechanisms independent of bacterial colonization, replication, and host–microbe competition. This approach is intended to facilitate mechanistic investigations and aligns with the emerging interest in postbiotic-based strategies for gut health support.

We hypothesized that Lr6127-derived metabolites protect epithelial barrier function and modulate immune responses under inflammatory stress, without necessarily inducing transcriptional remodeling of epithelial barrier-associated genes. To test this hypothesis, we employed an integrated in vitro approach combining epithelial permeability assays, gene expression analysis, stress-response marker measurements, cytokine profiling, and immune functional readouts following lipopolysaccharide challenge. By using canine epithelial and immune cell models, this study aimed to provide insights based on canine-derived epithelial and immune cell models into whether and how *L. reuteri*-derived metabolites can support intestinal homeostasis under inflammatory conditions and to advance the development of species-appropriate nutritional strategies for canine gut health. Studies investigating probiotic-derived metabolites in canine-specific epithelial and immune cell models remain limited, and standardized methodological frameworks for these systems are still emerging.

## 2. Results

### 2.1. Lr6127 Cell-Free Supernatant Mitigated Barrier Leakage

**Figure 1:**
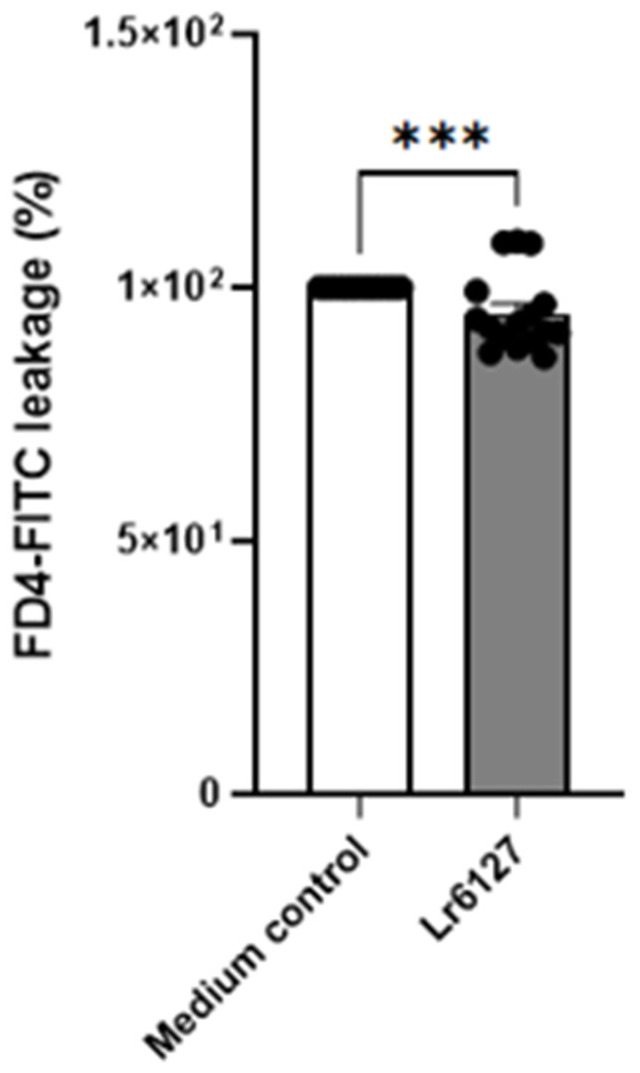
Effect of an Lr6127 cell-free supernatant (CFS) on epithelial barrier permeability in LPS-challenged MCA-B1 cells. Barrier permeability was assessed by measuring FD4-FITC leakage and is presented as a percentage relative to the LPS-treated-medium control, which was normalized to 100%. Data represent the mean ± standard error (SE) from two independent experiments with a total of 16 replicates per treatment group. Individual data points are shown. *** *p* < 0.001 (Lr6127 CFS vs. medium control).

Barrier permeability was assessed in MCA-B1 cells under LPS-challenge conditions using FD4-FITC leakage as a readout (Figure 1). In this experimental setting, treatment with an Lr6127 cell-free supernatant (CFS) resulted in a modest reduction in epithelial permeability compared with the medium control. Quantitatively, Lr6127 CFS decreased FD4-FITC leakage to 94.9 ± 1.9% (normalized relative to control levels set as 100%), corresponding to an average reduction of approximately 5% (*p* < 0.001). As permeability was normalized to the LPS condition, the observed changes reflect attenuation of LPS-induced barrier disruption rather than restoration to untreated baseline levels.

To further explore potential mechanisms, transcriptional markers associated with tight junctions and cellular stress responses were assessed in MCA-B1 cells. Tight junction-associated gene expression (Supplementary Figure S1) showed no transcriptional changes under the tested conditions. In addition, epithelial stress-related gene expression including oxidative stress-responsive enzyme heme oxygenase-1 (HMOX1) and HSP70is provided in Supplementary Figure S2, indicating modest transcriptional changes, with HMOX1 reaching statistical significance, and offering additional context for the observed permeability results.

### 2.2. Lr6127 Cell-Free Supernatant Modulated LPS-Induced Cellular Stress-Response Genes in DH82 Cells

**Figure 2:**
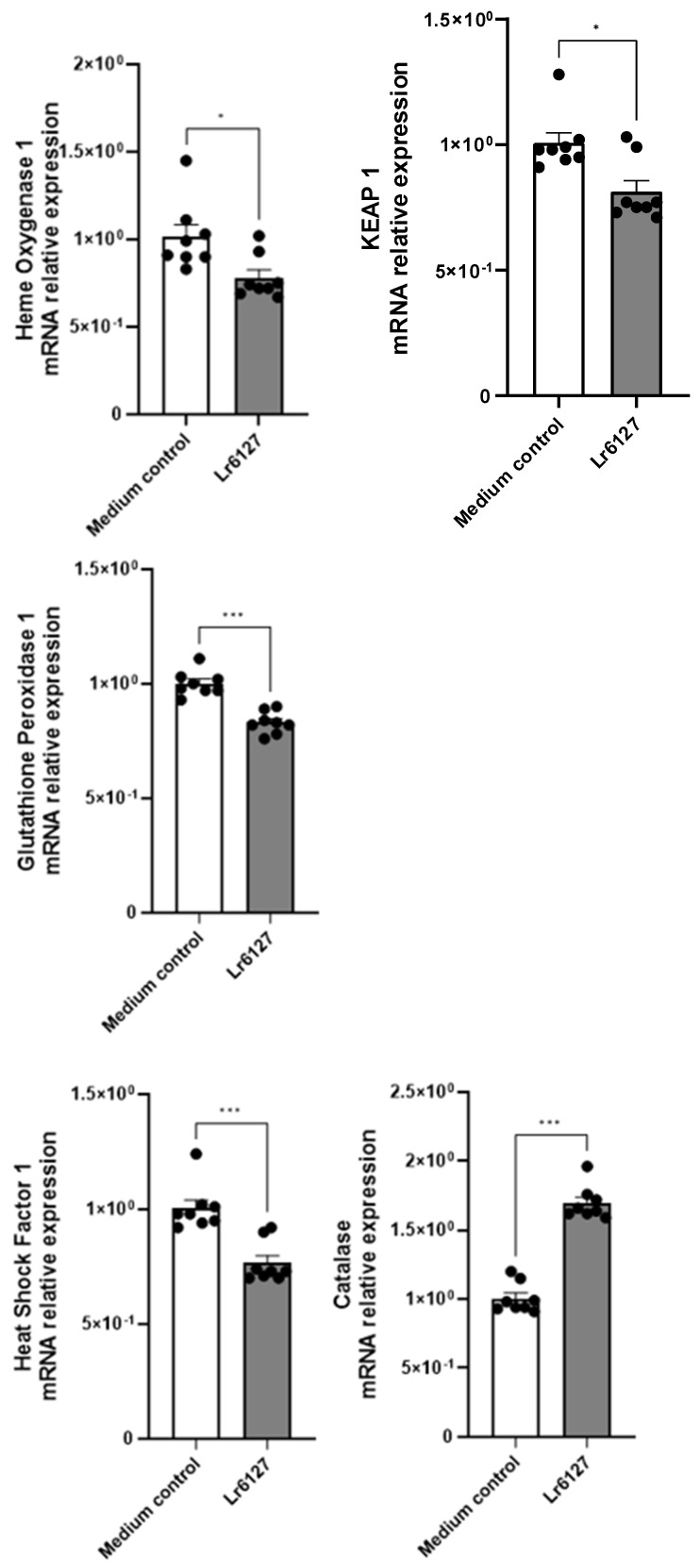
Effect of an Lr6127 cell-free supernatant (CFS) on the mRNA expression of heme oxygenase-1 (HMOX1), Kelch-like ECH-associated protein 1 (KEAP1), glutathione peroxidase 1 (GPX1), catalase, and heat shock factor 1 (HSF1) in LPS-treated DH82 cells. Gene expression levels were quantified by RT-qPCR and are presented as relative expression levels normalized to the expression levels of housekeeping genes (ACTB, GAPDH, and HPRT). Bars represent means ± SEs, with individual data points shown (*n* = 8 per treatment group). * *p* < 0.05, *** *p* < 0.001 (Lr6127 vs. medium control).

To determine whether Lr6127 cell-free supernatant (CFS) could influence LPS-induced stress-related gene expression patterns in immune-derived cells, the expression of stress- and redox-associated genes was examined in DH82 macrophage-like immune cells using RT-qPCR (Figure 2). Treatment with Lr6127 CFS resulted in coordinated changes in multiple biomarkers associated with cellular stress and antioxidant responses. mRNA levels of the oxidative stress-responsive enzyme heme oxygenase-1 (HMOX1) were reduced, decreasing from 1.0 ± 0.1 in the medium control to 0.8 ± 0.0 following Lr6127 CFS exposure (*p* < 0.05). Similarly, expression of Kelch-like ECH-associated protein 1 (KEAP1), a key regulator of redox-sensing pathways, declined from 1.0 ± 0.0 to 0.8 ± 0.0 (*p* < 0.05). The expression of glutathione peroxidase 1 (GPX1), an antioxidant enzyme that participates in peroxide detoxification, was also reduced, decreasing from 1.0 ± 0.0 in control cells to 0.8 ± 0.0 in Lr6127 CFS-treated cells (*p* < 0.001). In parallel, heat shock factor 1 (HSF1), a heat-shock response regulator, showed a similar decrease, with mRNA levels declining from 1.0 ± 0.0 to 0.8 ± 0.0 following treatment (*p* < 0.001). In contrast to these reductions, the expression of catalase, an antioxidant defense enzyme that catalyzes hydrogen peroxide clearance, was increased following Lr6127 CFS treatment, rising from 1.0 ± 0.0 in the medium control to 1.7 ± 0.0 (*p* < 0.001).

### 2.3. Lr6127 Cell-Free Supernatant Enhanced Anti-Apoptosis Gene Expression Under LPS-Induced Stress in DH82 Cells

**Figure 3:**
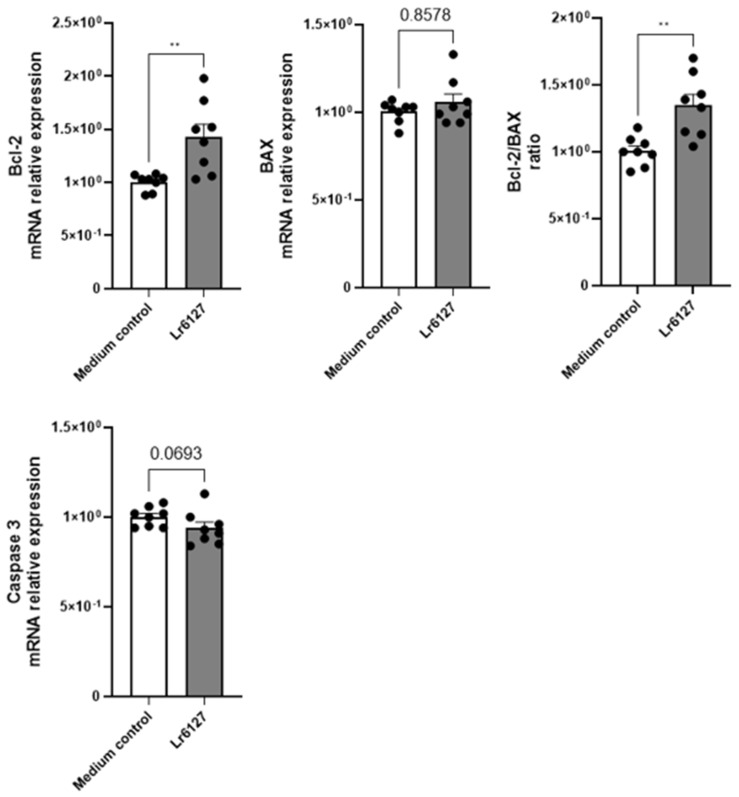
Effect of an Lr6127 cell-free supernatant (CFS) on the mRNA expression of B-cell lymphoma-2 (BCL2), BCL2-associated X protein (BAX), and caspase-3 (CASP3) and the BCL2/BAX ratio in LPS-treated DH82 macrophage-like immune cells. Gene expression levels were quantified by RT-qPCR and are presented as relative expression levels normalized to the expression levels of housekeeping genes (ACTB, GAPDH, and HPRT). Bars represent means ± standard errors (SEs), with individual data points shown (*n* = 8 per treatment group). Statistical significance is indicated where applicable; exact *p* values are shown for comparisons that did not reach significance. ** *p* < 0.01 (Lr6127 vs. medium control).

Given the coordinated attenuation of LPS-induced cellular stress and redox-associated gene expression observed in DH82 cells (Section 2.2), we next examined whether these stress-modulating effects were accompanied by changes in apoptotic gene expression, which could indicate an impact on cellular survival. The expression of apoptosis-related genes was therefore assessed in LPS-challenged DH82 cells using RT-qPCR (Figure 3). Treatment with an Lr6127 cell-free supernatant (CFS) selectively enhanced markers associated with cell survival. The anti-apoptotic regulator B-cell lymphoma-2 (BCL2) was significantly up-regulated, with its mRNA levels increasing from 1.0 ± 0.0 in the medium control to 1.4 ± 0.1 following Lr6127 CFS exposure (*p* < 0.01). In contrast, expression of the pro-apoptotic factor BCL2-associated X protein (BAX) remained unchanged between treatment groups (*p* = 0.8578). Consequently, the BCL2/BAX ratio, a commonly used indicator of the balance between pro- and anti-apoptotic signaling, was significantly elevated in Lr6127 CFS-treated cells, increasing from 1.0 ± 0.0 to 1.3 ± 0.1 (*p* < 0.01). Caspase-3 (CASP3), the executioner caspase, showed a modest downward trend following Lr6127 CFS treatment, although this change did not reach statistical significance (*p* = 0.0693).

### 2.4. Lr6127 Cell-Free Supernatant Attenuated LPS-Induced Pro-Inflammatory Gene Expression in DH82 Cells

**Figure 4:**
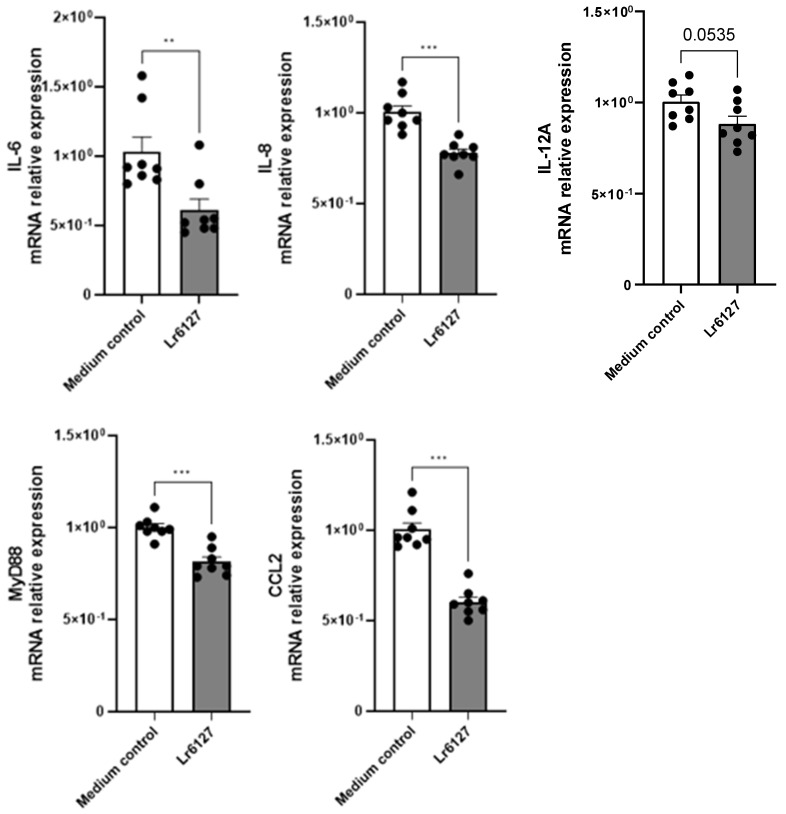
Effect of an Lr6127 cell-free supernatant (CFS) on the mRNA expression of interleukin-6 (IL-6), interleukin-8 (IL-8), interleukin-12A (IL-12A), myeloid differentiation primary response protein 88 (MYD88), and C-C motif chemokine ligand 2 (CCL2) in LPS-challenged DH82 macrophage-like immune cells. Gene expression levels were quantified by RT-qPCR and are presented as relative expression levels normalized to the expression levels of housekeeping genes (ACTB, GAPDH, and HPRT). Bars represent means ± SEs, with individual data points shown (*n* = 8 per treatment group). Statistically significant differences are indicated where applicable; IL-12A exhibited a non-significant downward trend (*p* = 0.0535). ** *p* < 0.01, *** *p* < 0.001.

To further assess whether the stress- and survival-modulating effects of the Lr6127 cell-free supernatant (CFS) were accompanied by altered inflammatory responses under LPS-challenge conditions, the expression of pro-inflammatory cytokines, chemokines, and signaling mediators was examined in DH82 cells using RT-qPCR (Figure 4). Treatment with Lr6127 CFS resulted in a coordinated attenuation of multiple biomarkers associated with LPS-induced inflammatory activation. The pleiotropic pro-inflammatory cytokine interleukin-6 (IL-6), which is rapidly induced downstream of Toll-like receptor signaling and contributes to amplification of inflammatory responses, was significantly down-regulated, with its mRNA levels decreasing from 1.0 ± 0.1 in the LPS-treated-medium control to 0.6 ± 0.1 following Lr6127 CFS exposure. Similarly, expression of interleukin-8 (IL-8), a chemotactic cytokine involved in the recruitment of neutrophils and other immune cells to sites of inflammation, was significantly reduced from 1.0 ± 0.0 to 0.8 ± 0.0. Expression of interleukin-12A (IL-12A), a cytokine subunit implicated in the regulation of pro-inflammatory and cell-mediated immune responses, exhibited a downward trend following Lr6127 CFS treatment but did not reach statistical significance. At the signaling level, the adaptor protein myeloid differentiation primary response protein 88 (MYD88)—a central mediator of LPS-triggered Toll-like receptor signaling and downstream cytokine induction—was significantly reduced from 1.0 ± 0.0 in control cells to 0.8 ± 0.0 in Lr6127 CFS-treated cells. In addition, the chemokine C-C motif chemokine ligand 2 (CCL2), which plays a key role in monocyte recruitment during inflammatory responses, was markedly down-regulated, decreasing from 1.0 ± 0.0 to 0.6 ± 0.0.

### 2.5. Proteomic Profiling Revealed Reduced Abundance of Pro-Inflammatory Proteins Under LPS Challenge in DH82 Cells Following Lr6127 CFS Treatment

**Figure 5:**
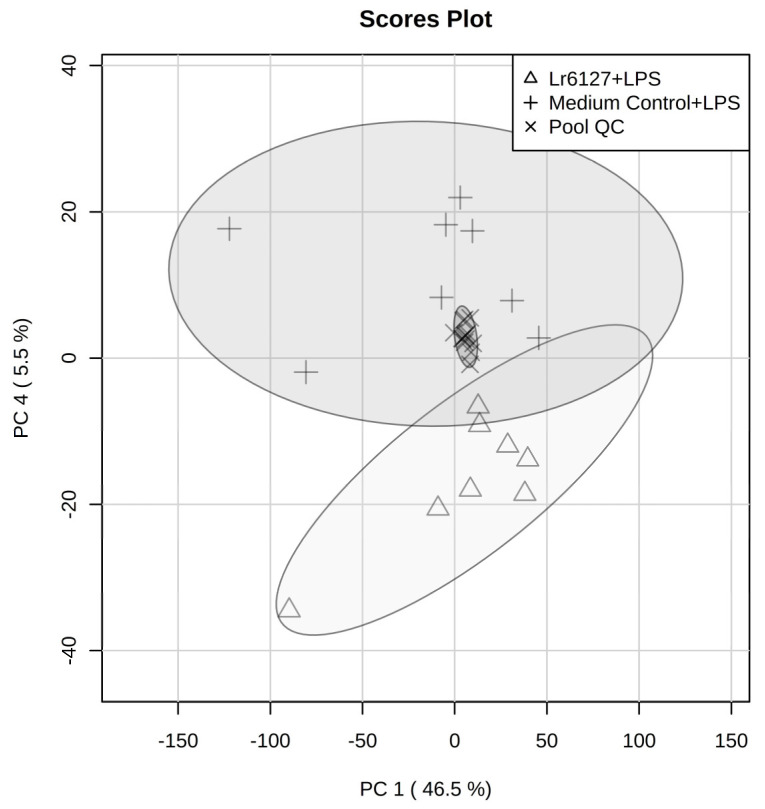
Principal component analysis (PCA) of proteomic data from LPS-challenged DH82 macrophage-like cells treated with control medium or Lr 6127 CFS. Each point represents an individual biological replicate analyzed by quantitative mass spectrometry. The samples separated according to treatment condition across the principal components, indicating global differences in protein expression profiles. Pooled quality control (QC) samples clustered in the center, confirming the analytical stability and reproducibility of the proteomic workflow.

To assess global proteomic alterations induced by Lr6127 cell-free supernatant (CFS) under inflammatory stress, an unsupervised principal component analysis (PCA) was first performed on the proteomic profiles of LPS-challenged DH82 cells (Figure 5). The PCA revealed a clear separation between the LPS-treated-medium control samples and the Lr6127 CFS-treated samples along principal component 4, indicating distinct global protein expression patterns between the two conditions. Pooled quality control (QC) samples clustered tightly near the center of the PCA space, confirming the analytical stability and reproducibility of the proteomic workflow.

**Figure 6:**
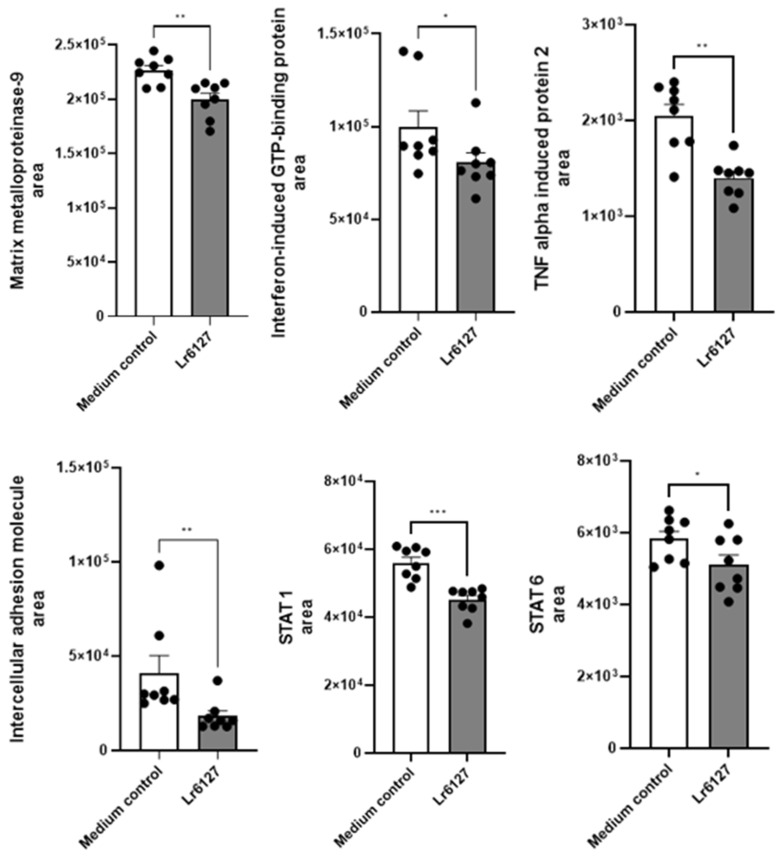
Proteomic analysis of LPS-challenged DH82 macrophage-like immune cells treated with an Lr6127 cell-free supernatant (CFS). Relative protein abundances of matrix metalloproteinase-9 (MMP-9), interferon-induced GTP-binding protein, tumor necrosis factor-alpha-induced protein 2 (TNFAIP2), intercellular adhesion molecule (ICAM), signal transducer and activator of transcription 1 (STAT1), and STAT6 are shown. Protein levels were quantified by mass spectrometry and are presented as abundance values derived from peak area intensity measurements. Bars represent means ± SEs, with individual data points shown (*n* = 8 per treatment group). Statistical significance is indicated as follows: * *p* < 0.05, ** *p* < 0.01, and *** *p* < 0.001.

Consistent with the PCA-based separation, quantitative proteomic analysis identified a coordinated reduction in the abundance of multiple proteins associated with inflammatory activation, immune cell recruitment, and cytokine-driven signaling pathways following the Lr6127 CFS treatment (Figure 6). Specifically, there was a reduced abundance of multiple proteins associated with inflammatory activation and immune signaling following the Lr6127 CFS treatment. The levels of matrix metalloproteinase-9 (MMP-9), a matrix-remodeling enzyme, was significantly reduced, decreasing from 2.3 ± 0.0 × 10^5^ in the LPS-treated-medium control to 2.0 ± 0.1 × 10^5^ following the Lr6127 CFS exposure (*p* < 0.01). Similarly, the abundance of an interferon-induced GTP-binding protein decreased from 1.0 ± 0.1 × 10^5^ to 0.8 ± 0.1 × 10^5^, corresponding to an approximately 19% reduction (*p* < 0.05). A marked reduction was also observed for tumor necrosis factor-alpha-induced protein 2 (TNFAIP2), whose abundance declined from 2.0 ± 0.1 × 10^3^ in the LPS-treated control to 1.4 ± 0.1 × 10^3^ following the Lr6127 CFS treatment (*p* < 0.01). In addition, protein levels of intercellular adhesion molecule (ICAM) were substantially reduced, decreasing from 4.1 ± 0.9 × 10^4^ to 1.8 ± 0.3 × 10^4^ (*p* < 0.01). Regarding inflammatory transcriptional regulators, signal transducer and activator of transcription 1 (STAT1) showed a significant reduction from 5.5 ± 0.4 × 10^4^ to 4.6 ± 0.1 × 10^4^ (*p* < 0.001), while STAT6 abundance decreased from 5.8 ± 0.2 × 10^3^ in LPS-treated cells to 5.1 ± 0.3 × 10^3^ following the Lr6127 CFS exposure (*p* < 0.05).

### 2.6. Proteomic Profiling Revealed Modulation of Immune-Related Proteins in Resting DH82 Cells Following Lr6127 CFS Treatment

**Figure 7:**
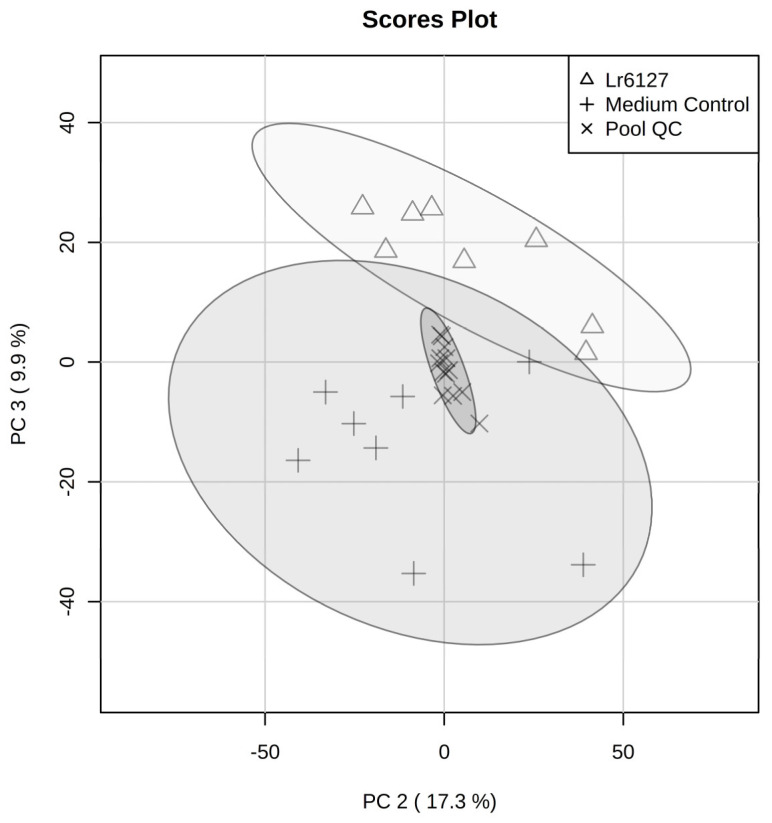
Principal component analysis (PCA) of proteomic data from resting (non-LPS-challenged) DH82 macrophage-like cells treated with medium or *L. reuteri* 6127 cell-free supernatant (CFS). Each point represents an individual biological replicate. The separation of treatment groups across principal components indicates distinct global protein expression patterns under basal conditions. Pooled quality control (QC) samples clustered tightly, demonstrating the consistency and robustness of the proteomic analysis.

To determine whether Lr6127 cell-free supernatant (CFS) modulates immune protein expression under basal conditions, PCA was performed on proteomic datasets derived from resting (non-LPS-challenged) DH82 cells (Figure 7). In contrast to the LPS-challenge condition, the PCA demonstrated a distinct separation between Lr6127 CFS-treated cells and medium control cells, reflecting systematic differences in protein expression despite the absence of inflammatory stimulation. Similarly to the results shown in Section 2.5, the pooled QC samples clustered tightly, indicating robust experimental consistency.

**Figure 8:**
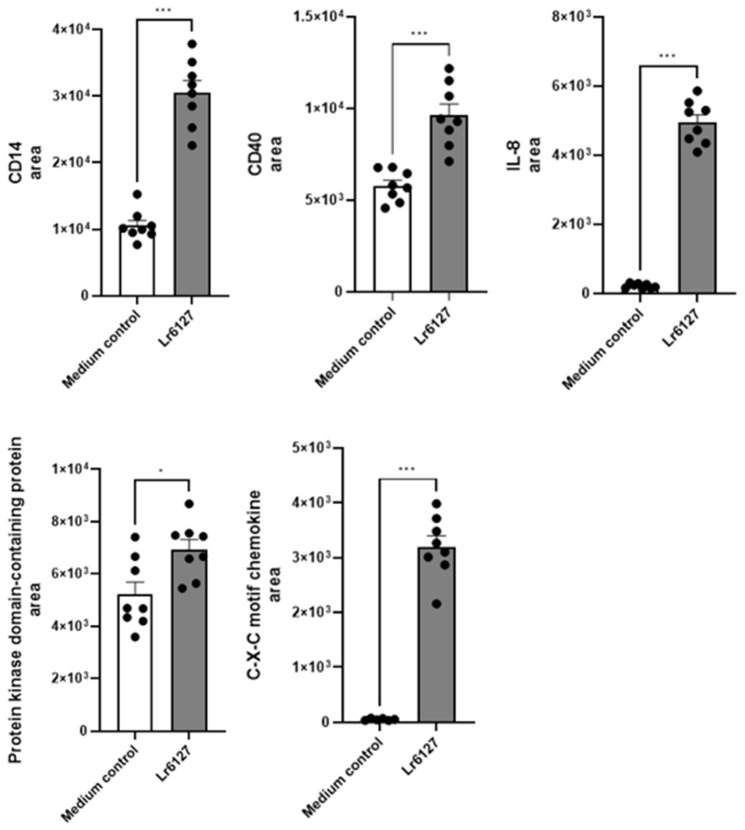
Proteomic analysis of resting (non-LPS-challenged) DH82 macrophage-like immune cells following treatment with an Lr6127 cell-free supernatant (CFS). Relative protein abundances of cluster of differentiation 14 (CD14), cluster of differentiation 40 (CD40), interleukin-8 (IL-8), protein kinase domain-containing protein, and C-X-C motif chemokine are shown. Protein levels were quantified by mass spectrometry and were derived from peak area intensity measurements. Bars represent means ± SEs, with individual data points shown (*n* = 8 per treatment group). Statistical significance is indicated as follows: * *p* < 0.05 and *** *p* < 0.001.

The subsequent quantitative analysis revealed increased abundances of several proteins involved in innate immune sensing, co-stimulatory signaling, and chemokine-mediated communication in Lr6127 CFS-treated resting cells (Figure 8). The abundance of cluster of differentiation 14 (CD14), a pattern recognition coreceptor that contributes to basal innate immune responses, increased from 1.1 ± 0.1 × 10^4^ in the medium control to 3.1 ± 0.2 × 10^4^ after Lr6127 exposure (*p* < 0.001). Similarly, cluster of differentiation 40 (CD40), a costimulatory receptor, exhibited an increased abundance, rising from 5.8 ± 0.3 × 10^3^ to 9.6 ± 0.6 × 10^3^ (*p* < 0.001), while the chemokine interleukin-8 (IL-8) showed a pronounced increase, with protein levels increasing from 2.2 ± 0.2 × 10^2^ in control cells to 4.9 ± 0.2 × 10^3^ following Lr6127 CFS treatment (*p* < 0.001). The protein kinase domain-containing protein, whose levels reflect intracellular signaling activity in steady-state immune cells, was modestly but significantly increased, rising from 5.2 ± 0.5 × 10^3^ to 6.9 ± 0.4 × 10^3^ (*p* < 0.05). In addition, the C-X-C motif chemokine displayed a marked increase in abundance, increasing from 5.1 ± 0.7 × 10^1^ in the medium control to 3.1 ± 0.3 × 10^3^ in Lr6127 CFS-treated cells (*p* < 0.001).

## 3. Discussion

In this study, we found that metabolites derived from *Limosilactobacillus reuteri* ATCC PTA 6127 (Lr6127), delivered as a cell-free supernatant (CFS), induced changes in epithelial and immune cell responses, especially under inflammatory stress induced by lipopolysaccharide (LPS). The use of a CFS enables the direct evaluation of probiotic-derived metabolites as functional bioactives, allowing for the investigation of host-directed mechanisms that operate independently of bacterial colonization or direct host–microbe contact. Using canine epithelial and macrophage-like cell models, our findings indicate that under an LPS challenge, Lr6127-derived metabolites reduced the expression of stress-response-related genes, increased indicators of cell survival, and modulated markers associated with immune-related pathway, which coincided with changes in epithelial barrier function and inflammatory responses. While the present findings were derived from a single *L. reuteri* strain and specific in vitro models, all transcriptional analyses were performed using validated assays specific for *Canis familiaris* targets, and proteomic data were analyzed against a canine database, ensuring that the observed responses reflect a canine-derived experimental system. Studies investigating probiotic-derived metabolites in canine-specific epithelial and immune cell models remain limited, and standardized methodological frameworks for these systems are still emerging. Therefore, the results should be interpreted considering this experimental context and should not be generalized to other strains or biological systems without further validation.

A notable finding of this study is that epithelial barrier permeability was reduced under LPS challenge despite the absence of detectable changes in epithelial barrier-associated gene expression (tight junction, adherens junction, or mucin genes). This observation is consistent with established models of intestinal barrier regulation, which propose that inflammatory barrier dysfunction is often driven by stress- and cytokine-dependent cytoskeletal remodeling rather than transcriptional down-regulation of tight junction component genes [14]. Structural analyses of tight junction biology further support that functional changes in paracellular permeability can occur without alterations in the abundance of junctional proteins [16]. In addition, probiotic-derived metabolites have been reported to preserve epithelial barrier integrity under inflammatory conditions without inducing tight junction gene expression, and instead they act through modulating inflammatory tone [17]. However, regulation of these genes at the protein level, including post-transcriptional, translational, or localization-dependent mechanisms, could also contribute to barrier function. Taken together, our observations support the view that epithelial barrier function is governed by integrated and multi-layered regulatory processes, rather than being solely dependent on transcriptional regulation of tight junction components or other structural elements such as adherens junction and mucin proteins. Within this framework, the observed reduction in permeability reflects a modest but reproducible attenuation of LPS-induced barrier disruption. Although limited in magnitude within this defined in vitro model, the consistency of this effect across independent experiments supports a measurable and reproducible response under the defined inflammatory conditions.

Considering the absence of transcriptional changes in epithelial barrier-associated genes, we next examined whether changes in stress-related transcriptional markers could provide an alternative interpretation for the reduced epithelial leakage. Lr6127 CFS resulted in changes in stress-associated gene expression, including a reduction in HMOX1, a stress-responsive transcriptional marker [18]. While this effect was limited in magnitude and not observed across all markers, it aligns with the broader pattern of cellular responses observed in this study. Similarly, the down-regulation of HMOX1 in LPS-treated DH82 cells is consistent with altered stress-response pathways [19]. These observed changes in stress-response marker transcription indicate which pathways are involved and offer a foundation for future studies integrating functional assessments of cellular stress. Reduced basal activation of stress-response pathways has been associated with improved cellular resilience rather than impaired cytoprotection [20]. Previous studies on probiotic metabolites have described antioxidant effects at the functional level [21]; however, the present results indicate an association between changes in stress-response gene expression and reduced epithelial permeability, providing a basis for future studies to directly investigate the causal relationships using functional stress assays.

Macrophage-like DH82 cells were a major focus of this study due to their central role in coordinating inflammatory responses and epithelial–immune crosstalk in the canine gut. In vivo, canine intestinal immune cells are continuously exposed to LPS derived from Gram-negative bacteria, particularly during dysbiosis or barrier impairment [22]. Under LPS-stimulated conditions, Lr6127-derived metabolites attenuated the expression of stress- and redox-associated genes while enhancing anti-apoptotic signaling. Comparable anti-inflammatory effects of probiotic metabolites have been reported in LPS-activated macrophage models, where metabolite exposure reduced inflammatory cytokine expression and oxidative stress responses [23]. Our findings extend these observations by showing that inflammatory attenuation is accompanied by increased immune cell survival, as reflected by the increased BCL2/BAX ratio, suggesting stabilization of immune cell function during inflammatory challenge. Although it did not reach statistical significance, CASP3 expression showed a consistent downward trend, which aligns with the overall pattern of apoptosis-related marker regulation. Taken together, these findings support a coordinated modulation of apoptosis-related pathways; further functional studies could further characterize the extent of this anti-apoptotic activity. In addition, the observed reductions in GPX1 and KEAP1 expression may reflect context-dependent biological responses, including potential attenuation of oxidative stress or adaptive modulation of immune activity, highlighting the dynamic nature of host–cell interactions elicited by Lr6127-derived metabolites.

An important strength of this study is the agreement between the transcriptional and proteomic readouts in LPS-challenged immune cells. Many previous studies investigating probiotic or postbiotic effects have predominantly relied on gene expression analyses, which may not capture sustained functional immune modulation [24]. Protein-level regulation of inflammatory signaling pathways, particularly STAT-associated pathways, has been identified as a critical determinant of immune-cell activation and resolution during endotoxin exposure [25]. The concordance observed in the present study between reduced inflammatory gene expression and decreased abundance of inflammatory proteins therefore supports durable modulation of immune signaling rather than transient transcriptional effects.

Notably, immune modulation by Lr6127-derived metabolites exhibited context-dependent patterns under inflammatory and resting conditions. Under LPS stimulation, the metabolites attenuated inflammatory responses, whereas under resting conditions, they increased the abundance of proteins involved in innate immune sensing, co-stimulatory signaling, and chemokine-mediated communication. This pattern may reflect enhanced cellular responsiveness or readiness for host defence, as molecules such as CD14, CD40, and IL-8 are involved in pathogen recognition and initiation of immune responses. These observations are from defined experimental settings and highlight the capacity of Lr6127-derived metabolites to differentially influence immune-related pathways depending on the cellular environment, consistent with the emerging evidence of context-dependent immunomodulation by probiotic-derived metabolites [25]. While direct within-condition comparisons were not included in the present study design, the consistent directionality of responses supports a context-dependent mode of action. In addition, the potential contribution of pattern recognition receptor activation, such as through TLR2- or NOD2-mediated signaling, cannot be excluded and may provide complementary insights into the observed modulation of immune-associated proteins under resting conditions.

Compared with prior studies emphasizing tight junction remodeling or isolated cytokine endpoints, this work highlights cellular stress regulation, redox balance, and apoptosis resistance as integrated mechanisms underlying epithelial and immune outcomes under inflammatory challenge. The regulation of cellular stress responses is being increasingly recognized as an upstream determinant of tissue homeostasis in models of host–microbiota interactions [21,26]. In this study, cellular stress responses were evaluated at the transcriptional level using established stress-responsive markers, providing insights into pathway engagement within this experimental context. The integration of complementary functional measurements, such as ROS levels, antioxidant enzyme activity, glutathione balance, phagocytosis, or nitric oxide production, would further refine the interpretation of these responses and should be pursued in future studies to support the translation of these findings into physiologically relevant settings. These observations are particularly relevant for canine gastrointestinal health as dogs are chronically exposed to LPS from their intestinal microbiota, and this exposure may increase during dietary changes, gastrointestinal infections, or chronic enteropathies [22]. Recent reviews emphasize the need for host-directed mechanisms to explain probiotic efficacy beyond bacterial colonization alone [27]. It should be noted that the magnitude of the observed effects in this in vitro model was modest and may not reflect the extent of barrier dysfunction or recovery observed under in vivo pathological condition. In this context, by modulating LPS-induced cellular stress, supporting immune balance, and contributing to epithelial barrier function, Lr6127-derived metabolites may therefore provide a basis for further investigation into their potential role in supporting intestinal homeostasis in vivo.

In addition to the above considerations, the experimental framework offers opportunities for further refinement in future studies. Expanding the control conditions, incorporating quantitative cytotoxicity assessments, and evaluating multiple LPS concentrations would further strengthen our proposed mechanism and help to better define the response sensitivity of epithelial and immune cells. Normalization based on CFU equivalents provides a practical and consistent framework for standardizing metabolite exposure across experiments; however, it does not capture absolute concentrations of individual metabolites, which may be further addressed in future targeted studies. To support the characterization of the cell-free supernatant, untargeted metabolomic profiling was performed, and PCA indicated no major differences in global metabolite profiles within this experimental framework. This is consistent with the concept that biological effects may be driven by specific signaling-active metabolites rather than large-scale compositional changes. Additional strategies such as defined metabolite controls or fractionation approaches could further refine the identification of active components and enhance mechanistic resolution in future studies.

In summary, this study demonstrated, using canine in vitro models, that Lr6127-derived metabolites are associated with measurable and reproducible effects on epithelial barrier function and host cellular responses under LPS challenge. The observed transcriptional and proteomic changes indicate involvement of stress- and inflammation-related pathways and are best interpreted as coordinated cellular adaptations within the experimental context rather than direct mechanistic effects. While the magnitude of individual responses was modest, their consistency across complementary experimental approaches supports a biologically relevant interaction between Lr6127-derived metabolites and host cells. These findings provide a foundation for further investigations incorporating functional assessments to better define the underlying mechanisms and physiological relevance.

## 4. Materials and Methods

### 4.1. Reagents and Materials

Unless stated otherwise, all cell culture media, reagents, and consumables were purchased from Thermo Fisher Scientific (Roskilde, Denmark).

### 4.2. Probiotic Strain and Preparation of Cell-Free Supernatant (CFS)

The probiotic strain *Limosilactobacillus reuteri* (ATCC PTA-6127; PureStrong™, Lr6127) was licensed from BioGaia AB (Stockholm, Sweden) and characterized by the supplier. Lr6127 was propagated anaerobically at 37 °C in de Man–Rogosa–Sharpe (MRS) agar and broth. Cell-free supernatant (CFS) was generated from bacterial cultures, whose growth was monitored by optical density measurements at 600 nm (OD_600_) using an EnSight Multimode Plate Reader (PerkinElmer, Shelton, CT, USA). The cultures were centrifuged to remove bacterial cells, and the supernatant was subsequently passed through sterile 0.2 µm vacuum filtration units (Thermo Scientific Nalgene aPES membrane, Thermo Fisher Scientific) and stored at −20 °C until further use.

### 4.3. Cell Line and CFS–Cell Coculture

The canine proximal epithelial cell line MCA-B1 (DSMZ, Braunschweig, Germany; ACC 828) and macrophage-like canine cell line DH82 (LGC Standards GmbH, Wesel, Germany; CRL-3590) were cultured in DMEM/F12 medium supplemented with 10% heat-inactivated fetal bovine serum (FBS) and 1% penicillin–streptomycin (100 U/mL penicillin and 100 µg/mL streptomycin) at 37 °C in a humidified atmosphere containing 5% CO_2_. Where indicated, lipopolysaccharide (LPS) was added at a final concentration of 100 ng/mL.

CFS was applied to cell cultures at a maximum concentration of 5% (*v*/*v*). Preliminary testing verified that this concentration did not affect the medium pH, which remained within the range of 7.0–7.2. Optical density measurements were correlated with viable cell numbers through serial dilution and colony counting, with an OD_600_ of 1.0 corresponding to approximately 1 × 10^9^ CFU/mL. All experiments were conducted using CFS at a working concentration equivalent to the bacterial secretions derived from 1 × 10^7^ CFU/mL as a standardized reference for bacterial input. This approach ensures consistency and reproducibility across experiments; however, as with CFU-based normalization strategies in cell-free systems, it reflects the overall metabolite exposure rather than absolute concentrations of individual compounds.

### 4.4. Permeability Assay

Paracellular permeability was evaluated using fluorescein isothiocyanate–dextran (FITC–dextran, 4 kDa; Merck Life Science A/S, Søborg, Denmark). MCA-B1 cells were seeded onto polyester Transwell inserts (surface area: 0.33 cm^2^; pore size: 0.4 µm; Corning, Corning, NY, USA) at a density of 2 × 10^5^ cells per insert and allowed to form confluent monolayers. When they reached confluency, the cells were either left untreated for 24 h or treated with cell-free supernatant (CFS) at a concentration equivalent to the bacterial secretions derived from 1 × 10^7^ CFU/mL. The monolayers were subsequently challenged with lipopolysaccharide (LPS, 100 ng/mL). This LPS concentration (100 ng/mL) was selected based on literature guidance [28] and prior experiments in comparable epithelial and macrophage-like in vitro systems, where it consistently induced measurable inflammatory and barrier responses without overt disruption of cell morphology or monolayer integrity. Control conditions consisted of the corresponding medium without Lr6127-derived components, including an equivalent volume of MRS, ensuring that basal medium effects were comparable between treatment groups. A formal dose–response assessment was not performed in the present study, and no dedicated cytotoxicity assay was included; however, no morphological changes, loss of confluency, or signs of monolayer disruption were observed during the experimental period. After an additional 24 h, the apical medium was replaced with FITC–dextran at a final concentration of 1 mg/mL. The basolateral medium was collected after 4 h of incubation, and fluorescence was quantified using a SpectraMax i3x plate reader (excitation 485 nm, emission 535 nm, Molecular Devices, CA, USA). FITC–dextran concentrations were determined by referencing a standard curve.

Permeability was expressed as a percentage relative to the LPS-treated controls according to the following equation:

Permeability (%) = (FD4_in test sample_/Mean FD4_in LPS-treated sample_) × 100

Permeability values were normalized to the LPS-treated condition to specifically assess attenuation of LPS-induced barrier disruption. The assay was performed as two independent experiments with eight biological replicates per condition, yielding a total of sixteen replicates per treatment group.

### 4.5. RT-qPCR Analysis of Cell Gene Expression

MCA-B1 and DH82 cells were seeded into 96-well culture plates at a density of 2 × 10^5^ cells/mL and grown to confluence at 37 °C. The cells were then exposed to cell-free supernatant (CFS) at a concentration corresponding to the bacterial secretions derived from 1 × 10^7^ CFU/mL, as determined by prior OD_600_ calibration. Control wells were treated with DMEM containing equivalent volumes of MRS medium without bacterial exposure; these served as medium controls to account for the potential effects of the culture conditions. Following treatment, the plates were incubated overnight under humidified conditions with 5% CO_2_.

After incubation, the cells were rinsed with phosphate-buffered saline (PBS) and lysed in 200 µL of RLT buffer (Qiagen, Aarhus, Denmark) for 30 min at 37 °C. Proteinase K was added to a final concentration of 100 µg/mL as part of the RNA preparation workflow to facilitate protein digestion and improve downstream nucleic acid purity. This step facilitates the removal of protein components, including nucleoprotein complexes, which may interfere with RT-qPCR performance. Total RNA was isolated, and reverse-transcription quantitative PCR (RT-qPCR) analysis was conducted by Biotest (Trige, Denmark) using the TaqMan assays listed in Supplementary Table S1. The TaqMan assays used in this study are specific for canine (*Canis familiaris*) targets, as indicated by the “Cf” assay identifiers, and were used in our previous studies using the same canine epithelial and macrophage-like cell models, and demonstrated robust and reproducible performance [29,30]. Quality control measures were implemented in accordance with the provider’s standard protocols, including the use of appropriate controls and verification of amplification specificity to ensure the robustness of the data generated and to confirm that RNA integrity and assay performance were not adversely affected.

The gene expression panel comprised cytokines (IL-6, IL-8, and IL-12A), signaling and transcriptional regulators (MyD88), apoptosis-related markers (BCL2, Bax, and caspase-3), and stress-associated biomarkers (heat shock protein 70, heme oxygenase 1, KEAP1, glutathione peroxidase 1, catalase, and heat shock factor 1). Each experimental condition was analyzed using eight biological replicates. Gene expression data were normalized against three reference housekeeping genes (Supplementary Table S1) using the following approach:

ΔCt = Ct_target − Ct_housekeeping. Relative gene expression was calculated as Fold change = 2^−(ΔCt_sample−ΔCt_control)^ using the 2^−ΔΔCt^ method.

### 4.6. Proteomic Analysis

Co-culture of MCA-B1 or DH82 cells with the cell-free supernatant (CFS) was performed as described above. Following incubation, the cells were rinsed with phosphate-buffered saline (PBS) and lysed using a buffer containing 5% SDS, 100 mM triethylammonium bicarbonate, and a protease inhibitor cocktail. Proteomic analysis was conducted according to the workflow described by Kadekar et al. [31].

Peptide samples were analyzed using a Vanquish Neo ultra-high-performance liquid chromatography (UHPLC) system coupled to a Q Exactive HF mass spectrometer (Thermo Fisher Scientific, Bremen, Germany). Separation was performed in a trap-and-elute configuration employing a NanoViper trap column in combination with a nanoEase M/Z Peptide BEH C18 analytical column (Waters, Milford, MA, USA). A 70 min chromatographic gradient was applied at a flow rate of 2000 nL/min. Mass spectrometric data were acquired in data-dependent acquisition (DDA) mode using higher-energy collisional dissociation (HCD) for peptide fragmentation.

Raw mass spectrometry data were processed using Proteome Discoverer version 3.0 and searched using the Mascot search engine against the *Canis familiaris* UniProt FASTA database. Each experimental condition was analyzed using eight independent biological replicates.

### 4.7. Statistical Analysis

Non-proteomic datasets were analyzed using non-parametric methods due to the distribution characteristics of the data. As comparisons were limited to two independent groups within each experimental setup, the Mann–Whitney U test was applied, with statistical significance defined as *p* < 0.05. The proteomic results were subjected to multivariate latent class analysis (LCA), after which pairwise comparisons were performed using *t*-tests with the false discovery rate correction set at 5%. Data processing and statistical analyses were performed using GraphPad Prism (v9).

## Data Availability

The data supporting the findings of this study are included within the article and its Supplementary Materials. Additional datasets generated and/or analyzed during the current study are available from the corresponding authors upon reasonable request.

## Supplementary Materials

The following supporting information can be downloaded at: https://www.mdpi.com/article/10.3390/ijms27125546/s1.

## Abbreviations

The following abbreviations are used in this manuscript:

CCL2	C-C motif chemokine ligand 2
CFS	Cell-free supernatant
DH82	Canine macrophage-like cell line
FITC-dextran	Fluorescein isothiocyanate–dextran
GPX1	Glutathione peroxidase 1
HMOX1	Heme oxygenase-1
HSF1	Heat shock factor 1
HSP70	Heat shock protein 70
IL	Interleukin
IL-6	Interleukin-6
IL-8	Interleukin-8
IL-12A	Interleukin-12 subunit alpha
KEAP1	Kelch-like ECH-associated protein 1
LPS	Lipopolysaccharide
MCA-B1	Canine intestinal epithelial cell line
MYD88	Myeloid differentiation primary response protein 88
NRF2	Nuclear factor erythroid 2-related factor 2
STAT1	Signal transducer and activator of transcription 1
STAT6	Signal transducer and activator of transcription 6
