# Supplementary material for: Barrier and Immune Modulation by Limosilactobacillus reuteri ATCC PTA 6127 in Canine Epithelial and Immune Cells Under Lipopolysaccharide Challenge
Source: Int J Mol Sci. 2026 Jun 19;27(12):5546. doi: 10.3390/ijms27125546 (PMC13299922; doi:10.3390/ijms27125546)
Supplement: Supplementary file 1 [file ijms-27-05546-s001.zip › ijms-4352300-supplementary.pdf]

Supplementary Results

Lr6127 Cell-Free Supernatants Had No Significant Effect on Epithelial Barrier–Associated Gene Expression in MCA-B1 Cells

To assess whether the observed reduction in epithelial permeability was associated with transcriptional changes in epithelial barrier components, the mRNA expression of epithelial barrier–associated genes was analyzed in MCA-B1 cells following exposure to Lr6127 cell-free supernatants. Treatment with Lr6127 cell-free supernatants did not result in significant changes in the expression of zonula occludens-1, claudin-1, claudin-4, E-cadherin, or mucin-1 compared with the medium control. For all genes analyzed, relative mRNA expression levels remained comparable between treatment groups, indicating that Lr6127-mediated changes in epithelial permeability were not associated with transcriptional modulation of epithelial barrier–associated genes.

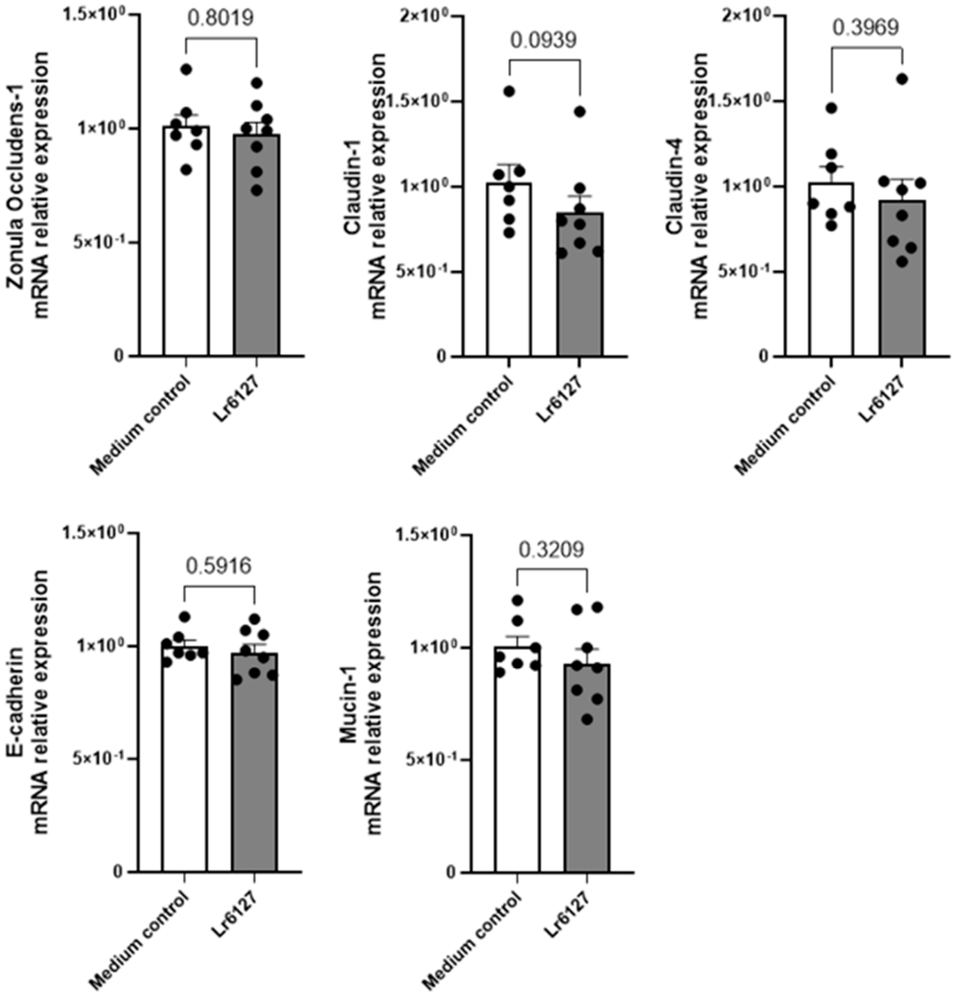

Figure S1. Effect of Lr6127 cell-free supernatants (CFS) on the mRNA expression of epithelial barrier–associated genes in MCA-B1 cells. Relative mRNA expression of zonula occludens-1, claudin-1, claudin-4, E-cadherin, and mucin-1 was quantified by RT-qPCR and normalized to medium control levels. Bars represent means ± SE, with individual data points shown. No statistically significant differences were observed between groups ($P > 0.05$).

Lr6127 Cell-Free Supernatants Reduced Oxidative Stress-Responsive Enzyme Heme Oxygenase-1 (HMOX1) Gene Expression in MCA-B1 Cells

Given the observed reduction in epithelial permeability following treatment with an Lr6127 cell-free supernatant (CFS), the expression of molecular markers associated with epithelial barrier regulation and cellular stress responses was measured in MCA-B1 epithelial cells using RT-qPCR. Notably, despite the reduction in epithelial permeability, the Lr6127 CFS treatment did not alter the mRNA expression of classical epithelial barrier components, including ZO-1, claudin-1, claudin-4, mucin-1, or E-cadherin (Supplementary Figure S1). These findings suggest that the reduced barrier leakage was not associated with transcriptional changes in tight junction, adherens junction, or mucin genes.

Next, transcriptional markers associated with cellular stress responses were assessed in MCA-B1 cells. (Figure S2). Compared with the medium control, Lr6127 CFS treatment resulted in lower mRNA expression of the oxidative stress-responsive enzyme heme oxygenase-1 (HMOX1), which decreased from 0.9 ± 0.1 in control cells to 0.7 ± 0.1 following Lr6127 CFS exposure ($P < 0.05$). A decreasing trend in the expression of heat shock protein 70 (HSP70), a molecular chaperone involved in cellular protection against protein-folding stress, was also observed.

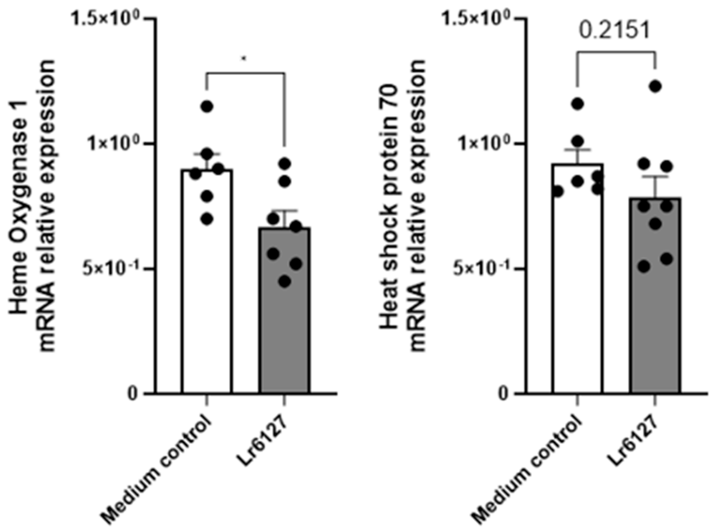

Figure S2. Effect of an Lr6127 cell-free supernatant (CFS) on the mRNA expression of heat shock protein 70 (HSP70) and heme oxygenase-1 (HMOX1) in MCA-B1 cells. Gene expression levels were quantified by RT-qPCR and are presented as relative expression levels normalized to the expression levels of housekeeping genes (ACTB, GAPDH, and HPRT). Bars represent means ± SEs, with individual data points shown. * $P < 0.05$ (Lr6127 CFS vs. medium control).

Table S1. Taq Man ID numbers and housekeeping genes used in the RT-qPCR.

**Genome target in MCA-B1 cells**	**TaqMan ID No.**
Heat Shock Protein 70	Cf02698320_g1
Heme Oxygenase-1	Cf02626655_m1
Kelch-like ECH-associated protein 1	Cf01003432_m1
Glutathione Peroxidase 1	Cf02731517_s1
Heat Shock Factor 1	Cf02678699_m1
Catalase	Cf02621925_m1
Bcl-2	Cf02622425_m1
BAX	Cf02727746_g1
Occludin	Cf02624089_m1
Caspase 3	Cf02622236_m1
IL-6	Cf02624153_m1
IL-8	Cf02624262_m1
IL-12	Cf02690011_m1
IL-18	Cf02624262_m1
MyD88	Cf02675965_m1
CCL2	Cf02741747_mH
ZO-1	Cf01552709_m1
Claudin-1	Cf02713195_u1
Claudin-4	Cf02695489_s1
E-cadherin	Cf02697525_m1
Mucin-1	Cf02680908_s1
**Housekeeping genes**	
ACTB	Cf04931159_m1
GAPDH	Cf04419463_gH
HPRT	Cf02690456_g1
